# Correlation between low skeletal muscle index and 3D anthropometric data measured by 3D body scanner: screening sarcopenia

**DOI:** 10.3389/fmed.2024.1296418

**Published:** 2024-02-22

**Authors:** Kyu Wan Kim, Yongsoo Park, Yohan Lee, Minjoon Cho

**Affiliations:** ^1^Department of Orthopedic Surgery, Seoul National University Boramae Hospital, Seoul, Republic of Korea; ^2^Medi Help Line Inc., Seoul, Republic of Korea; ^3^Department of Orthopedic Surgery, Seoul National University College of Medicine, Seoul, Republic of Korea

**Keywords:** sarcopenia, skeletal muscle index, bio-impedance analysis, screening, 3D scanner, 3D anthropometry

## Abstract

**Background:**

The screening tools for sarcopenia are measuring calf circumference, SARC-F or SPPB. However, not all of these tools have high sensitivity, specificity, and low margins of error. This research investigates potential of 3D anthropometry of the lower extremities on screening of sarcopenia.

**Methods:**

From October 2022 to February 2023, we retrospectively analyzed results of 3D body scanner and bio-impedance analysis for patients aged 45 to 85 at risk of sarcopenia. The 3D scanner measured the surface and volume values of both thighs and calves. When skeletal muscle index (SMI) is less than 5.7, patients were classified to Low SMI group, indicative of sarcopenia.

**Results:**

A total six out of 62 patients were classified to Low SMI group, showing significantly lower values of right, left, mean calf volumes and mean calf surface than the other patients (right calf volume 2.62 L vs. 3.34 L, *p* = 0.033; left calf volume 2.62 L vs. 3.25 L, *p* = 0.044; mean calf volume 2.62 L vs. 3.29 L, *p* = 0.029; mean calf surface 0.12 m^2^ vs. 0.13 m^2^, *p* = 0.049). There was no statistical difference in thigh volume and surface. Through AUC-ROC analysis, mean calf volume was the most significant cut-off value (right calf volume 2.80 L, AUC = 0.768; left calf volume 2.75 L, AUC = 0.753; mean calf volume 3.06 L, AUC = 0.774; mean calf surface 0.12 m^2^, AUC = 0.747).

**Conclusion:**

The calf volume and surface values have significant relationship with low SMI, and the mean calf volume was the most significant cut-off screening value for Low SMI. The 3D scanner demonstrated its value as a new means for screening sarcopenia.

## Introduction

Sarcopenia, characterized by the age-related loss of skeletal muscle mass and functional decline, has emerged as a significant health concern in the aging population (1). Sarcopenia is associated with limitations in independent living, diminished physical performance, and increased risks of acute and chronic diseases, ultimately decreasing quality of life and health (2).

The early diagnosis of sarcopenia is crucial for appropriate management and treatment (3). However, the accurate diagnosis of sarcopenia remains challenging, as measurement methods and assessors may vary, allowing sarcopenia to be undiagnosed until significant loss of skeletal muscle mass and functional decline have occurred. The core issue lies in the initial stage of detection and screening processes. Therefore, various screening tools and diagnostic tests have been developed to aid in the identification and confirmation of sarcopenia.

The calf muscle circumference can be measured in the initial screening of sarcopenia, with a cut-off of 34 cm or less for males and 33 cm or less for females (Asian Working Group for Sarcopenia (AWGS) guidelines) (4). The Strength, Assistance with walking, Rising from a chair, Climbing stairs, and Falls (SARC-F) questionnaire and the Short Physical Performance Battery (SPPB), consisting of balance, gait speed, and chair-stand tests that assess specific risk factors, symptoms, or functional limitations associated with sarcopenia, are also used for screening (5, 6).

While these tools are not invasive, easy to administer, provide a quick initial assessment, and help to determine the need for further diagnostic evaluation, they may not always be sufficiently sensitive or specific, leading to potential false negatives or positives, and having disadvantages to use as screening tools (7).

Additionally, diagnoses are based on quantitative muscle measurements with assessments of muscle performance in everyday life. However, current screening tools dominantly assess muscle function and inadequately assess muscle quantity (6). The superiority of any particular tool remains a topic of debate (7–11). Therefore, a diagnosis of sarcopenia may be delayed until muscle mass loss and functional decline have already progressed, limiting the effectiveness of interventions and treatments and negatively impacting patients’ quality of life and independent function (12).

We considered using three-dimensional (3D) body scanning as an alternative to calf circumference measurement as a screening tool for evaluating muscle mass and volume in patients with sarcopenia. With the recent advancements in 3D depth camera technology, cameras have become small enough to be integrated into smartphones, their capture speed has increased, and they have become more common. Rapid 3D body measurements have become feasible, enabling 3D reconstruction of the craniofacial skeleton, teeth and teeth atlas, and even forensic applications in the medical field (13).

3D body measurements provide accurate and comprehensive physical measurements, offering values for the volume and surface area of various body parts. To the best of our knowledge, the 3D body scanner has not been previously studied for measuring skeletal muscle mass reductions. In this study, we analyzed the correlation between Skeletal Muscle Index (SMI) values measured by Bio-electrical Impedance Analysis (BIA) with a female sarcopenia cut-off value of 5.7 and limb volume and surface area values measured by a 3D body scanner.

## Methods

### Materials

This study was approved by the Institutional Board of Seoul National University Boramae Hospital (IRB No. 10-2022-114), and the requirement for patient consent was exempted. This was a single-center retrospective analysis of 3D body scanner measurements (Medi Help Line Co., Seoul, South Korea) and BIA analysis of patients who visited our hospital from October 2022 to February 2023. The patients came for health check-ups, and the BIA and 3D body scans were being conducted free of charge. The BIA and 3D body scanner that were previously available for free use at fitness centers or health centers, had been acquired by our hospital’s health screening center. Subsequently, the examinations were conducted targeting patients who volunteered through our outpatient clinic banner advertisements.

### 3D body scanning

**Figure 1:**
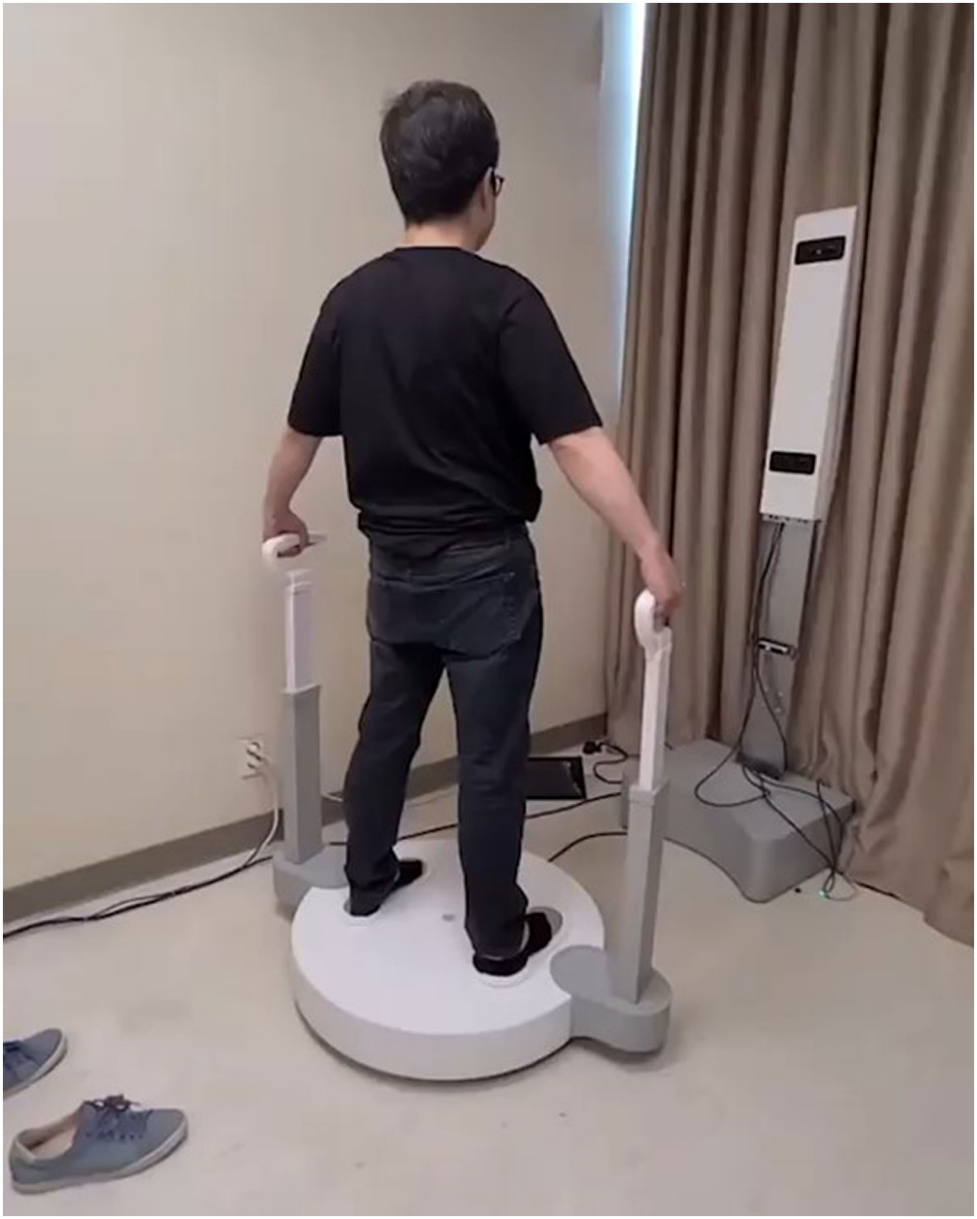
An example of poses for 3D scanner imaging.

**Figure 2:**
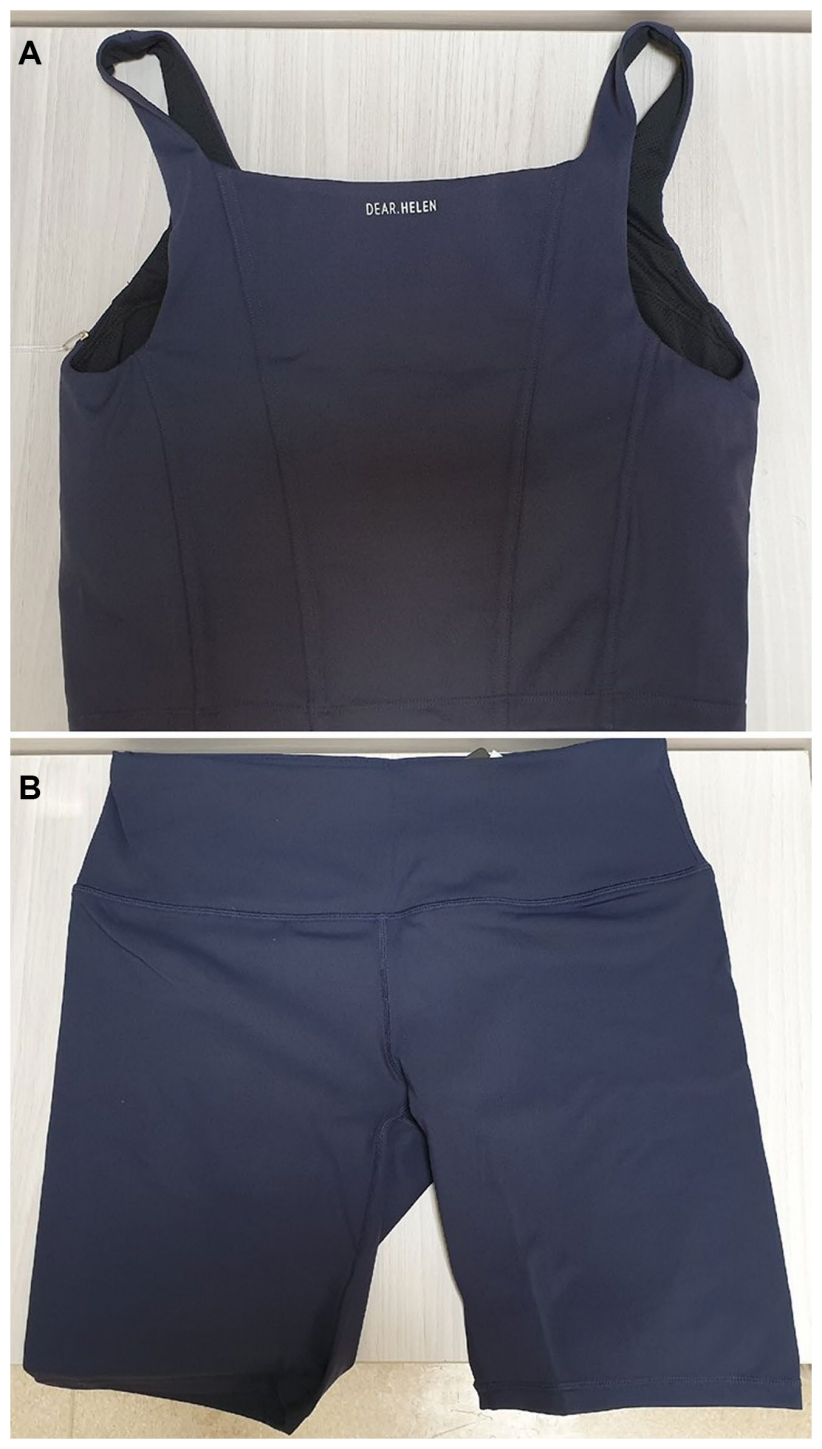
Activewear that adheres to the body was worn during 3D scanner imaging. **(A)** Top, **(B)** Shorts.

The 3D body scanner measured the surface area and volumes of chest, pelvis, upper extremities, both thighs and calves. The results of 3D body scans can vary due to factors such as the patient’s posture and clothing. Therefore, we standardized the scanning procedure as follows:

1. Posture: The position of the patient being scanned can significantly affect the results. Generally, a standard standing pose is adopted, with feet shoulder-width apart, arms slightly raised, and looking forward at a fixed point at eye level. This posture enables a comprehensive 3D body scan, including every aspect of the lower extremities (Figure 1).
2. Clothing: Any clothing or accessories worn by patients being scanned can interfere with the scanner’s ability to accurately capture the body’s surface. Typically, wearing minimal clothing and accessories, including jewelry, watches, and accessories, that be removed is recommended. Patients were asked to wear form-fitting clothing provided by our department to ensure the most accurate body shape capture. For consistency, all patients in this study were scanned wearing the same type of clothing. Any accessories or bulky items were removed prior to the scan (Figure 2).
3. Body Movement: Body scanning requires the subject to remain completely still throughout the process. Any movement can distort the measurements.
4. Several measurements: If the patient moved during the measurement or the measurement was considered inappropriate for other reasons, the measurement was repeated until it was satisfactory.

### Participants

Female patients between the ages of 45 and 85 were included in this study to identify screening cut-off values to exclude gender difference bias. Patients with a history of malignant neoplasia and musculoskeletal disorders, with metal implants in the limbs due to orthopedic surgery or other reasons, were excluded. Patients’ medical histories were also considered. Individuals with conditions that could cause lower limb swelling, such as heart or kidney disease, varicose veins, lymphedema, lumbar radiculopathy, or lower extremity trauma within the past 6 months, and conditions that could potentially influence 3D body scanner measurements or BIA were excluded.

### Methods

We calculated the SMI value using BIA and height. SMI values were calculated by dividing the skeletal muscle mass (total muscle mass of four extremities) by the square of the height. The unit was kilograms per square meter (kg/m^2^). SMI values are commonly used as an indicator of muscle mass relative to body size and used in the assessment of sarcopenia and muscle-related conditions. When the SMI value is less than 5.7, the patient is considered to have low skeletal muscle mass, which might indicate sarcopenia. We classified these patients into the Low SMI group and the other patients to the control group.

### Statistical analysis

Mann-Whitney U test tests were used to compare nonparametric continuous variables, and Fisher’s exact test was used to compare categorical variables between groups. Statistical analyses were performed using Rex (http://rexsoft.org/, Version 3.0.3, RexSoft Inc., Seoul, Korea), an Excel-based statistical analysis software. The *p*-value < 0.05 was considered statistically significant. Continuous variables were presented as the mean (standard deviations) for parametric data and the median (interquartile range) for nonparametric data. Categorical variables were presented as number of patients (percentage). The factors assessed were age, body mass index (BMI), comorbidities (presence of hypertension, dyslipidemia, diabetes mellitus and osteoporosis), and the surface and volume of body parts.

## Results

### Characteristics of participants

A total 62 patients were included in the total group, and 6 patients were classified into the Low SMI group, whom BMI was less than 5.7. The mean age of the entire patients was 65.85 years, with a mean age of 66 years in the Low SMI group and 65.84 years in the control group; however, these differences were not statistically significant. The BMI was 24.9, 21.65 and 25.05, respectively. The comorbidities of the patients (hypertension, diabetes, hyperlipidemia, and osteoporosis) were collected; however, there were no statistically significant associations (Table 1).

**Table 1:** Comparison of anthropometric data between sarcopenic and non-sarcopenic patients.

Variables	Group			*p*-value^a^
	Total (*n* = 62)	Low SMI (*n* = 6)	Control (*n* = 56)	
Age	65.85 ± 6.58	66 ± 5.18	65.84 ± 6.75	0.9462^a^
BMI (kg/m^2^)	24.9 (23.13, 26.05)	21.65 (20.05, 23.55)	25.05 (23.75, 26.25)	0.019^a^
Comorbidities				
Hypertension	19 (30.65%)	2 (33.33%)	17 (30.36%)	>0.99^b^
Dyslipidemia	14 (22.58%)	2 (33.33%)	12 (21.43%)	0.61^b^
Diabetes mellitus	11 (17.74%)	1 (16.67%)	10 (17.86%)	>0.99^b^
Osteoporosis	12 (19.35%)	1 (16.67%)	11 (19.64%)	>0.99^b^
Thigh volume (ℓ)				
Right	3.95 (3.46–4.66)	4.01 (3.47–4.14)	3.92 (3.48–4.70)	0.5281^a^
Left	3.68 (3.29–4.35)	3.62 (3.30–3.86)	3.68 (3.30–4.41)	0.4533^a^
Mean	3.78 (3.37–4.51)	3.82 (3.37–4.00)	3.78 (3.38–4.57)	0.4824^a^
Thigh surface (m^2^)				
Right	0.11 (0.1–0.13)	0.12 (0.10–0.12)	0.11 (0.10–0.13)	>0.99^a^
Left	0.11 (0.1–0.12)	0.11 (0.11–0.11)	0.11 (0.10–0.12)	0.9336^a^
Mean	0.11 (0.1–0.13)	0.11 (0.11–0.12)	0.11 (0.10–0.13)	0.9525^a^
Calf volume (ℓ)				
Right	3.29 (2.97–3.59)	2.62 (2.52–3.01)	3.34 (3.03–3.69)	0.0331*^a^
Left	3.16 (2.79–3.43)	2.62 (2.49–2.72)	3.25 (2.88–3.45)	0.0442*^a^
Mean	3.23 (2.88–3.55)	2.62 (2.51–2.98)	3.29 (2.98–3.63)	0.0294*^a^
Calf surface (m^2^)				
Right	0.14 (0.13–0.15)	0.13 (0.12–0.13)	0.14 (0.13–0.15)	0.0843^a^
Left	0.13 (0.12–0.14)	0.11 (0.11–0.12)	0.13 (0.12–0.14)	0.0801^a^
Mean	0.13 (0.12–0.14)	0.12 (0.11–0.13)	0.13 (0.12–0.15)	0.0495*^a^

SMI, skeletal muscle index. Values are expressed as the mean ± standard deviation or median (interquartile range) for continuous variables, and number of patients (percentage) for categorical variables.

a Derived with Mann-Whitney U test.

b Derived with Fisher’s exact test.

*p-value < 0.05.

### Results in 3D body scan

The Low SMI patients showed statistically significantly lower right, left, and mean calf volumes than the control group (right calf volume, 2.62 ℓ vs. 3.34 ℓ, *p* = 0.033; left calf volume, 2.62 ℓ vs. 3.25 ℓ, *p* = 0.044; mean volume of both calves, 2.62 ℓ vs. 3.29 ℓ, *p* = 0.029). Also, the mean calf surface area was significantly lower than in the control group (mean surface of both calves, 0.12 m^2^ vs. 0.13 m^2^, *p* = 0.049). However, there was no statistical difference between the two groups in volume or surface area of the other body parts (Table 1).

**Figure 3:**
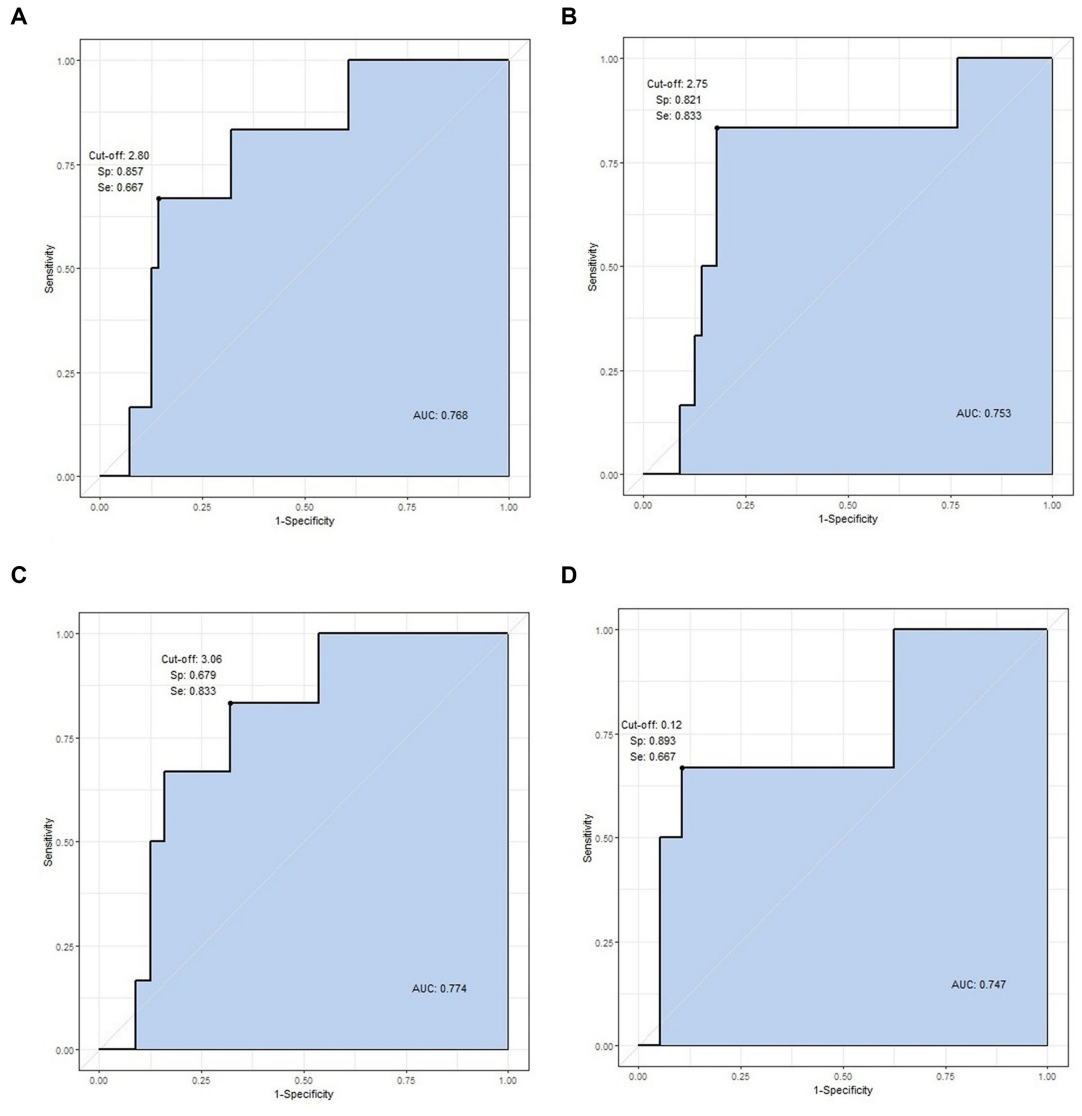
ROC curve analysis of statistically significant variables. The four values are cut-off value, specificity, sensitivity, and AUC, respectively. **(A)** Right calf volume (2.80, 0.857, 0.667, and 0.768 L), **(B)** left calf volume (2.75, 0.821, 0.833, and 0.753 L), **(C)** mean calf volume (3.06, 0.679, 0.833, and 0.774 L), and **(D)** mean calf surface area (0.12, 0.893, 0.667, 0.747 m^2^).

Area under the curve-receiver operating characteristic (AUC-ROC) analysis was conducted for four statistically significant values: right and left calf volume, mean calf volume, and mean calf surface area. The cut-off value for the right calf volume was 2.80 L, and the AUC value was 0.768 (specificity = 85.7%, sensitivity = 66.7%). The cut-off value was 2.75 L for the left calf volume, and the AUC value was 0.753, with the highest specificity and sensitivity (specificity = 82.1%, sensitivity = 83.3%). The cut-off and AUC values for the mean calf volume were 3.06 L and 0.774 (specificity = 67.9%, sensitivity = 83.3%), and for the mean calf surface area, the values were 0.12 m^2^ and 0.747, respectively (specificity = 89.3%, sensitivity = 66.7%). The mean calf volume of 3.06 L had the highest AUC value (a “fair” value of 0.774) and could be used as the most significant cut-off value (Figure 3). The specificity and sensitivity were highest for left calf volume.

## Discussion

Rapid advancements in 3D scanning and printing technologies capabilities have occurred, particularly over the past decade. 3D scanners have the advantages of high accuracy, high speed, easy manipulation, and low operational costs relative to computed tomography and magnetic resonance imaging (14–16). Moreover, there is minimal risk to the human body, such as radiation exposure, resulting in very few limitations on imaging and making it possible to perform scans outside of hospitals and in everyday spaces.

Many studies have reported applications in medical fields, apart from areas directly related, such as engineering and computer science (13). Research on 3D scanning applications, especially in fields such as dentistry, maxillofacial surgery, and plastic surgery, has been conducted (17, 18). While these prior studies were conducted by 3D scanning of specific body parts, research on full-body 3D scanning has yet to be conducted.

Full-body scanning data is already being collected as big data in some countries. In South Korea, the Ministry of Trade, Industry, and Energy has conducted a Korean anthropometric survey called “Size Korea” since 1979. Direct measurements using a tape measure have been conducted since 1979, and data on 103,254 people have been accumulated, while data on 15,429 people have been collected using a 3D scanner since 2003 and are freely accessible.^1^ In the United Kingdom, a survey called “SizeUK” measured 5,500 men and 5,500 women to create a national anthropometric database (19). The United States Centers for Disease Control and Prevention (CDC) periodically compiles and publishes anthropometric data on Americans (20).

Such data collection initiatives were originally commenced due to interests in fields related to body sizing, custom clothing, and virtual shopping. Yet, the potential for medical research based on these data cannot be overlooked. The significance of this study lies in establishing a foundation that associates sarcopenia with whole-body anthropometric data acquired through 3D body scanning. This research showed a correlation between sarcopenia and 3D anthropometry by setting cut-off values for calf volume and surface areas in patients with low SMI values (less than 5.7).

However, this study had the following limitations. First, this study did not target patients who were definitively diagnosed with sarcopenia. Additional tests on grip power, physical activity, or DEXA scans are required to diagnose sarcopenia. However, conducting these tests was impossible due to the retrospective study design. Therefore, it is imperative to conduct further research on the 3D anthropometry of patients diagnosed with sarcopenia, especially when integrated with functional assessments, such as grip strength, walking speed, and the SPPB.

Secondly, this study was based on the BIA test results of a small number of patients. However, BIA test results have high variability. Thus, the reliability of these values decreases when based on a small patient group. Therefore, research involving a larger number of patients or based on more reliable tests, such as DEXA scans instead of BIA results, is necessary. Lastly, this study targeted a single gender. While it is true that sarcopenia has a higher prevalence in women, research targeting both genders is required for demographic universality.

## Conclusion

Mean calf volume was the most useful 3D scanning result in women aged 45–85 with a low SMI value. The 3D scanner demonstrated its value as a new means for screening sarcopenia.

## Data availability statement

The datasets presented in this study can be found in online repositories. The names of the repository/repositories and accession number(s) can be found at: doi: 10.17632/4ps56b29tm.1.

## Ethics statement

The studies involving humans were approved by Seoul Metropolitan Government-Seoul National University Boramae Medical Center Institutional Review Board. The studies were conducted in accordance with the local legislation and institutional requirements. The ethics committee/institutional review board waived the requirement of written informed consent for participation from the participants or the participants’ legal guardians/next of kin because this study is a retrospective investigation conducted based on the information obtained during health check-ups of patients.

## Author contributions

KK: Writing – original draft, Writing – review & editing. YP: Conceptualization, Data curation, Investigation, Writing – review & editing. YL: Conceptualization, Formal analysis, Investigation, Methodology, Writing – original draft, Writing – review & editing. MC: Conceptualization, Investigation, Methodology, Project administration, Writing – review & editing.

## Footnotes

^1^http://sizekorea.kr
